# OECs transplantation results in neuropathic pain associated with BDNF regulating ERK activity in rats following cord hemisection

**DOI:** 10.1186/1471-2202-14-80

**Published:** 2013-08-02

**Authors:** Bing-Chen Lang, Zhuo Zhang, Long-Yun Lv, Jin Liu, Ting-Yong Wang, Ling-Hui Yang, Da-Qing Liao, Wen-Sheng Zhang, Ting-Hua Wang

**Affiliations:** 1 Regenerative Medicine Research Center, West China Hospital, Sichuan University, Chengdu, China; 2 Laboratory of Anesthesia and Critical Care Medicine, Translational Neuroscience Center, West China Hospital, Sichuan University, Chengdu, Sichuan 610041, P.R. China; 3 Institution of Neuroscience, Kunming Medical College, Kunming, China; 4 Department of Neurology, Tangdu Hospital, Fourth Military Medical University, Xi’an, China; 5 Institution of Neurological Disease, Translational Neuroscience Center, West China Hospital, Sichuan University, Chengdu, Sichuan 610041, P.R. China

**Keywords:** Olfactory ensheathing cells, Spinal cord injury, Hemisection, Cell transplantation, Rat, p-ERK, BDNF, Hyperalgesia

## Abstract

**Background:**

The olfactory ensheathing cells (OECs) derived from olfactory bulb (OB) may improve motor function after transplantation in injured spinal cord. However, the effects of OEC transplantation on sensory function have not been reported yet. The purpose of this study is to investigate whether OEC transplantation could affect the sensory function and to analyze the underlying mechanism.

**Results:**

OEC transplantation into the hemisected spinal cords can result in hyperalgesia, indicated by radiant and mechanical stimuli towards the plantar surface in rats. This could be associated with upregulation of Brain Derived Neurotrophic Factor (BDNF), indicated by RT-PCR. Immunofluorecent staining showed that BDNF was mainly located in the neurons of the laminas I and II of the dorsal horn. Moreover, a notable upregulation on the level of p-ERK (phosphorylation of extracellular signal-regulated kinase), the downstream molecule of BDNF, was detected by using Western Blot. These findings indicate that the increased BDNF level associated with the p-ERK was possibly involved in neuropathic pain in hemisected spinal cord subjected to OEC transplantation.

**Conclusions:**

The transplantation of OECs may induce the noticeable pain hypersensitivity in rats after hemisected spinal cord injury, and the possible mechanism may be associated with the phosphorylation of ERK and the activated BDNF overexpression.

## Background

To make the neuronal regeneration to eventual functional recovery after spinal cord injury (SCI), various treatments including surgical, pharmacological, and neurophysiologic approaches have been performed. Especially, cell transplantation with hypotoxicity and high effectiveness could be a promising strategy for the treatment of SCI.

As a special type of neuroglial cells derived from the olfactory bulb, olfactory ensheathing cells (OECs), are featured the permanent function of neural regeneration. Compared with the Schwann cells, the OEC possess higher migration ability and higher ability of integration with nerve axons and other neuroglial cells [1-3]. Reportedly, OECs can secrete various neurotrophins such as platelet derivation growth factor (PDGF), brain-derived neurotrophic factor (BDNF) [4,5], nerve growth factor (NGF) [6] and neurotrophin-4/5 [7]. In an injured spinal cord, the transplanted OECs have a neuroprotective effect on descending cortical and brain stem neurons [8,9], suggesting the effect of OECs on the improvement of motor function. However, it is still unknown whether the treatment of OEC transplantation could work well on the sensory function.

In this experiment, the mechanical and thermal hyperalgesia behaviors of rats with hemisected spinal cord injury (hSCI) were observed after OEC transplantation. Then we explored the underlying mechanism, which would be correlated with the phosphorylation of ERK and the up-regulation of BDNF.

## Results

### Identification of OECs in vitro

**Figure 1:**
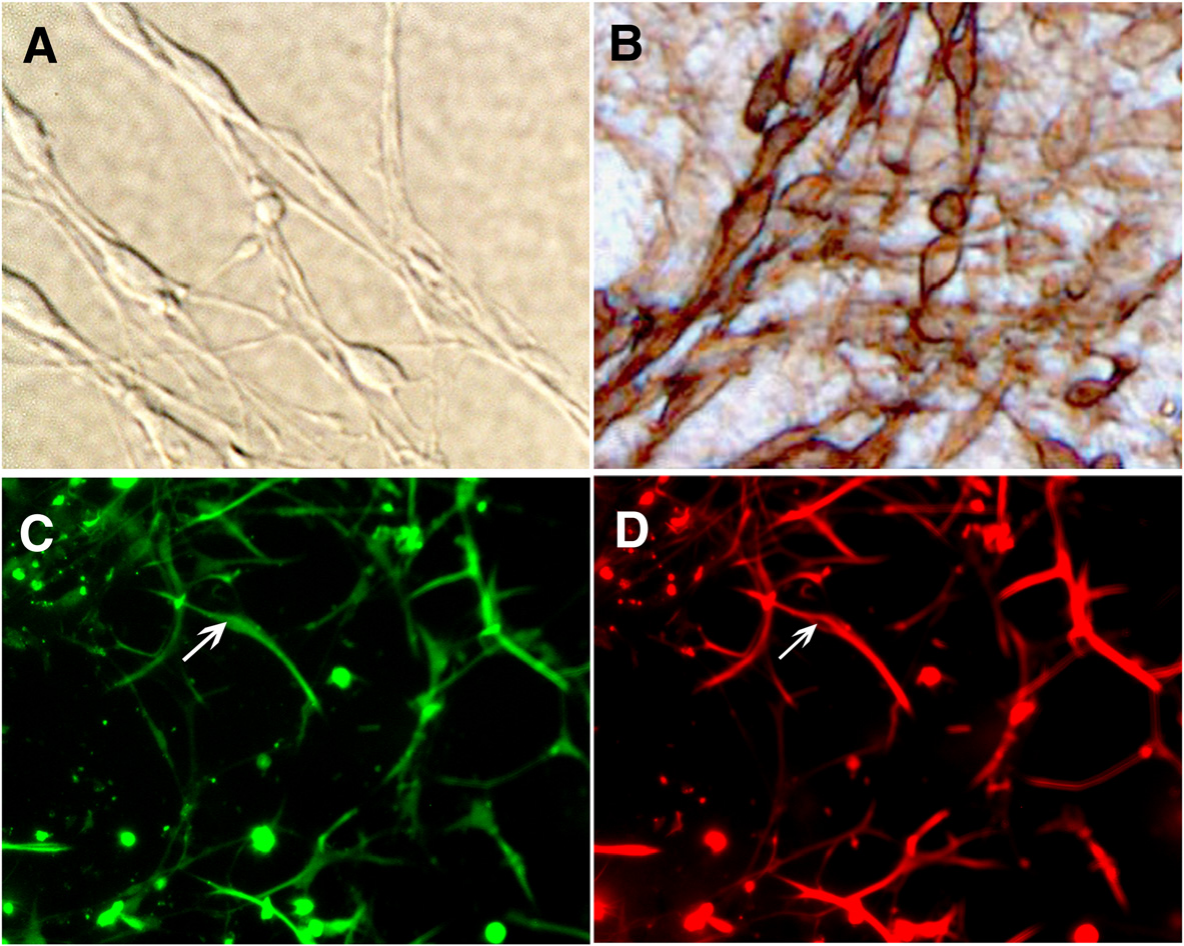
**Identification of OECs in vitro.** The cultured OECs were shown in **A**. The cells exhibited positive brown staining for neurotrophin surface receptor p75 (**B**, Brown staining with DAB). The GFP-OECs cells which emitting green fluorescence were seen in **C**, simultaneously, the red fluorescence confirmed that the GFP cells can express p75 **(D)**.

After 24 h of culturing, the adherent cells were in rounded shape and uniform distribution. After 3 days of culture, the cell bodies were amplified obviously and most of them formed triangles with two or three processes. After 5–7 days of culture, the characteristics of OECs *in vitro* were shown, and the cell processes were elongated remarkably and they contact mutually to form a network (Figure 1A). Enzyme immunochemical staining of the cultured cells were positive for neurotrophin p75 surface receptor, identified as OECs. The purity of OECs is higher than 95%. These cells were brown as a DAB reaction product was present in these cells (Figure 1B). In addition, OECs derived from the GFP transgenic mice could emit green fluorescence (Figure 1C). Based on red fluorescent staining under incubation by p75 antibody, we also identified the cultured cells as OECs *in vitro* (Figure 1D).

### Evaluation on Nociceptive tests

In sham-operated rats, the paw withdrawal latency (PWL) and paw withdrawal thresholds (PWT) were recorded separately.

#### Thermal stimuli

**Figure 2:**
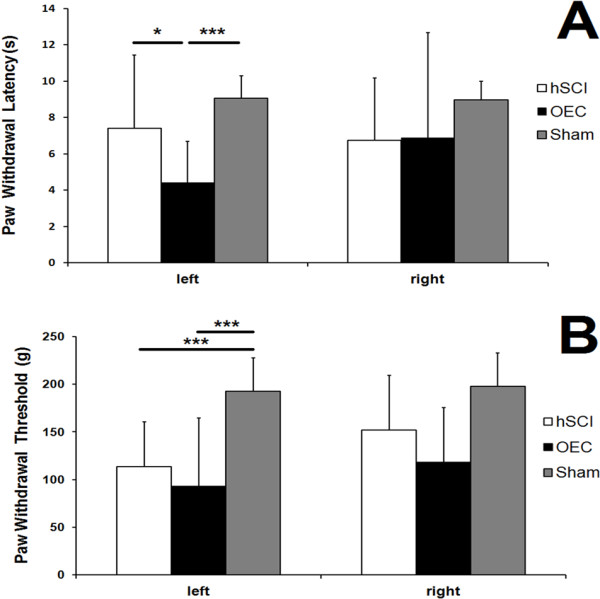
**Evaluations on Nociceptive Tests.** In the nociceptive evaluation of thermal stimuli, these asterisks indicate that the left paw withdrawal latency (PWL) of the OEC transplantation group was significantly lower than those of the injury group (*P < 0.05) and the sham group (***P < 0.001) **(A)**. In the nociceptive evaluation of thermal stimuli, the left paw withdrawal thresholds (PWT) of sham group were significantly higher than those of the injury group (***P < 0.001), while the PWTs of the OEC transplantation group were significant lower than the sham group (***P < 0.001) **(B)**.

In the nociceptive evaluation of thermal stimuli, these asterisks indicate that the left PWL of the OEC transplantation group (4.41 ± 2.38 s) was significantly lower than those of hSCI group (7.42 ± 4.20s) (*P < 0.05) and the sham group (9.06 ± 1.28 s) (***P < 0.001). There was no significant difference in left PWL between the sham group and the hSCI group. The quantitative analysis is shown in Figure 2A.

#### Mechanical stimuli

By using the pressure stimuli expressed by grams to the injury-side hind paws of rats in each group, we found that the PWTs of sham group (192.67 ± 39.25 g) were significantly higher than those of the hSCI group (113.33 ± 49.24 g) (***P < 0.001), and the OEC treatment group (93.33 ± 74.63 g) exhibited the lowest level (***P < 0.001). The quantitative analysis is shown in Figure 2B.

### Survival and migrating of OECs in vivo

**Figure 3:**
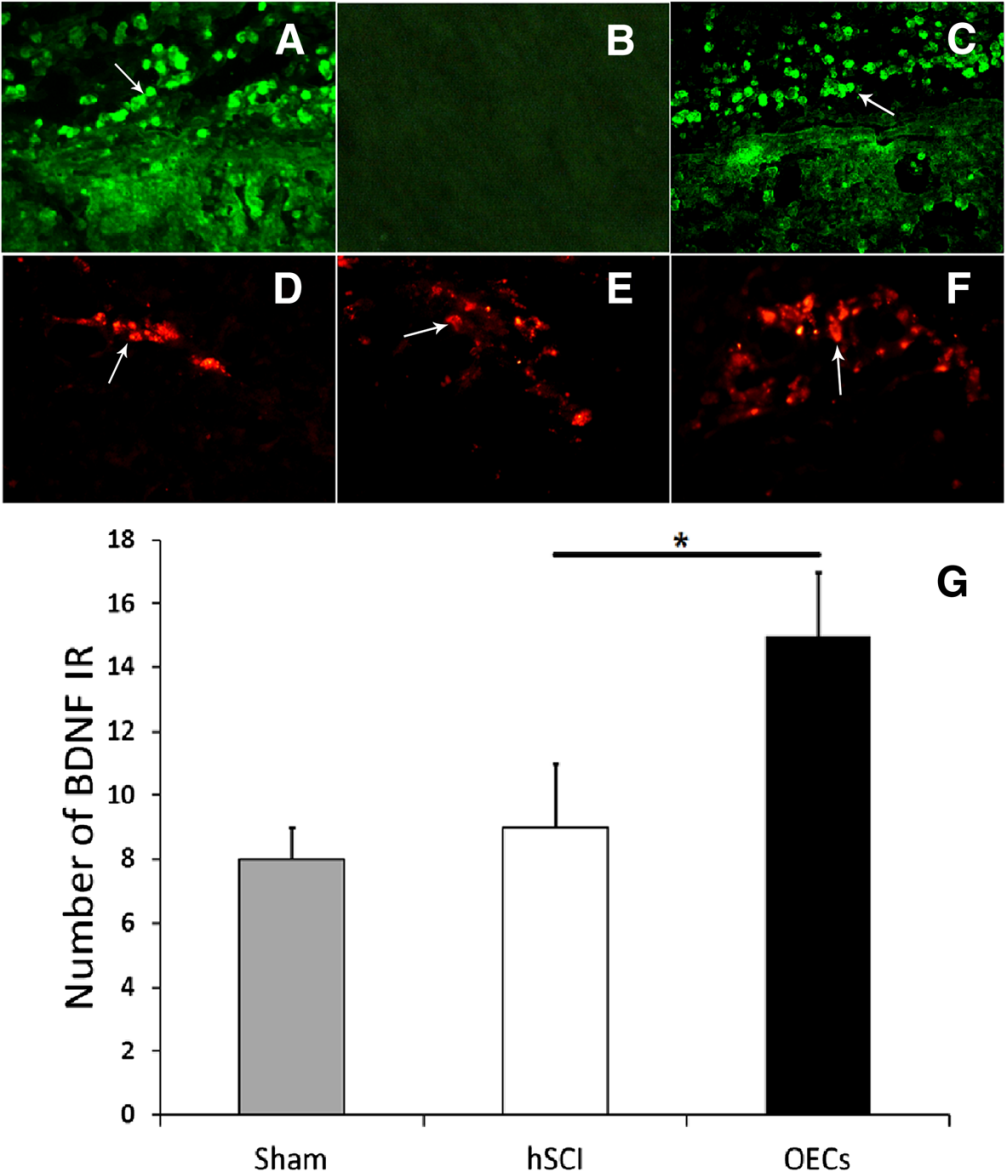
**Survival and migrating of OECs *in vivo*, and localization of BDNF.** Compared with the situation of injured control group **(B)**, implanted OECs had the capability of surviving in the injured spinal cord after implantation **(A)**. The OEC with green fluorescence around the site of injury were showed in **C**, indicating the transplanted cells could survive and migrate in spinal cord till one month after injury. Simultaneously, neurons in the dorsal horn could express BDNF,which emit red fluorescence **(D-F)**. The quantitative analysis was showed in **G**.

To confirm the survival of OECs with spinal cord injury, GFP (green fluorescent protein)-positive OECs were observed. As shown in Figure 3A and C, GFP-labeled OECs with fluorescence were seen in the spinal cord. The results confirmed that the implanted OECs were capable of surviving and migration in the spinal cord till one month after injury, compared with the situation of the hSCI group without cells transplantation (Figure 3B).

### BDNF co-expression in engrafted OECs and host neurons

In order to determine the change of BDNF-positive cells, we performed the immunofluorescent staining by using BDNF antibodies. The secondary antibodies were conjugated to the fluorescent marker Cy-3, which emits red fluorescence to be observed by fluorescent microscope. Compared with the sham group (Figure 3D) and the hSCI group (Figure 3E), the number of BDNF-positive cells was increased in the OECs transplantation group (Figure 3F). The quantitative analysis on the comparison of the numbers of BDNF-positive cells is shown in Figure 3G, which shows significant difference between the OECs transplantation group and the hSCI group (*P < 0.05).

The images indicated BDNF-positive cells were present not only in OECs engrafted with GFP fluorescence (Figure 3F), but also in neurons in the superficial layer of dorsal horn surrounding the spinal cord injury.

### BDNF co-expression in engrafted OECs and host neurons

**Figure 4:**
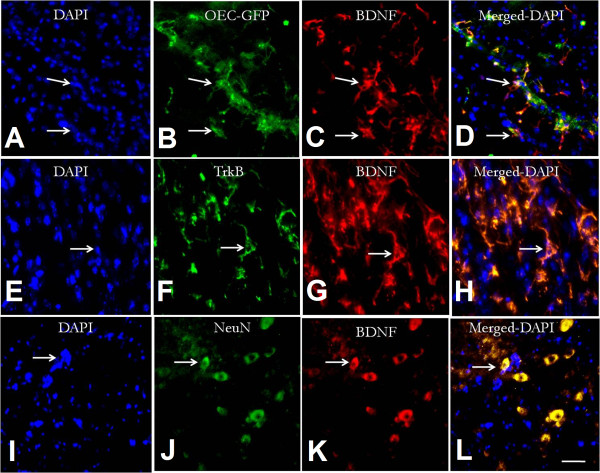
**Identification of BDNF expression in transplanted OECs and host neurons.** The distribution of nucleus would be showed in DAPI staining. It is showed that OECs can express BDNF after transplantation **(A-D)**. Meanwhile, the images also showed that the BDNF expression was concomitantly occurred in neurons **(E-H)**, while these neurons has co-expresseion of TrkB receptor **(I-L)**.

To identify the co-localization of BDNF in host neurons and engrafted OECs, the immunofluorecent double-label staining was performed by using anti-BDNF, anti-NeuN (neuronal marker), and anti-TrkB separately. Double-label staining showed that the engrafted OECs can express BDNF after transplantation (Figure 4A-4D). BDNF-positive cells were labeled simultaneously by anti-NeuN. This confirmed that the cells labeled by BDNF staining are neurons in the host spinal cord (Figure 4E-4H). In addition, the neurons labeled by BDNF were also found to express TrkB, the functional receptor of BDNF (Figure 4I-4L).

### The mRNA changes of BDNF in RT-PCR

**Figure 5:**
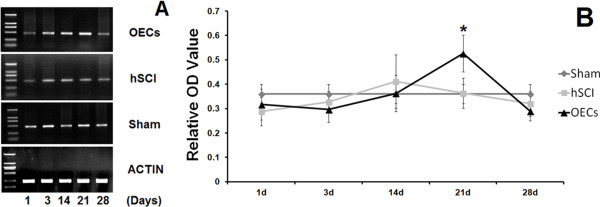
**mRNA changes of BDNF in RT-PCR.** OECs transplantation could largely upregulate the BDNF expression on the 21th day ininjured spinal cord after OEC transplantation **(A)**. The quantitative analysis was showed in **B**, indicating the significant difference of Relative OD Value between hemisected group and OECs transplantation group (*P < 0.05).

The mRNA expressions for BDNF were detected in the sham group, the hSCI group and the OECs transplantation rats. Significant difference between the hSCI group and the OEC transplantation group was detected on the 21st day (*P < 0.05), but not at other time points. Therefore, OEC transplantation could significantly upregulate the BDNF expression in the spinal cord after injury (Figure 5A, Figure 5B).

### Changes of ERK and p-ERK expressions detected by Western blot

**Figure 6:**
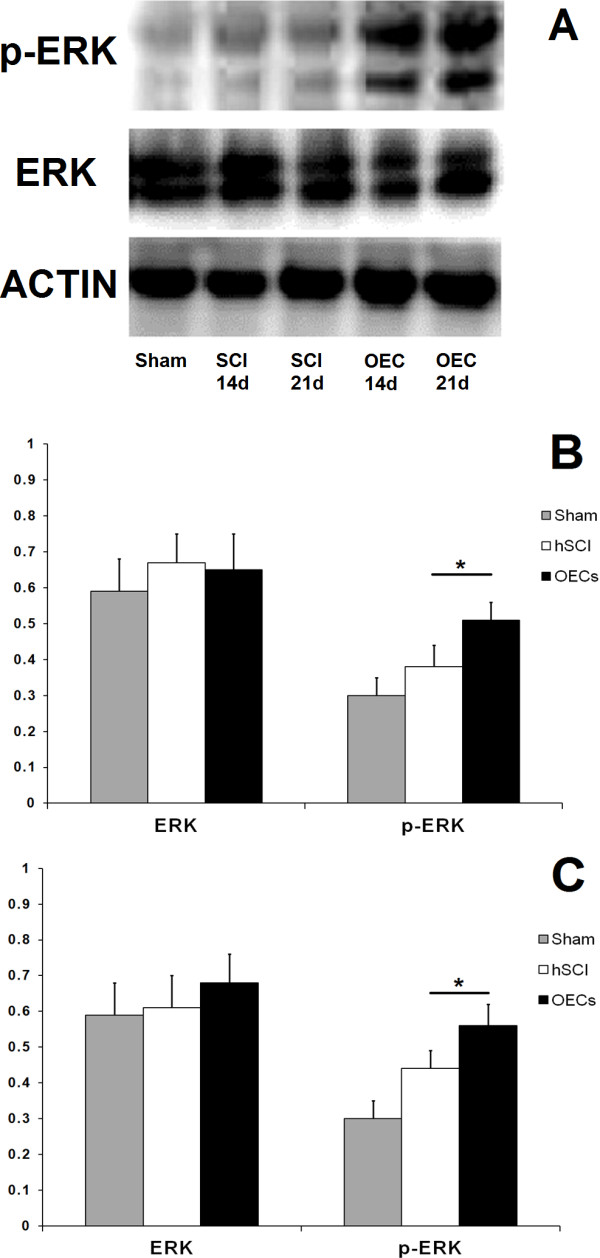
**Changes of ERK and p-ERK in OEC treated rats.** Compare with the sham group and the injury group, OECs graft can lead to a significant increase on the p-ERK expression both on the 14th day and the 21st day while there were no differences on the ERK expression among three groups **(A)**. The quantitative analyses for ERK and p-ERK on the 14th day and the 21st day were showed in **B**-**C**. The images indicated that the p-ERK expression increase significantly (*P < 0.05) on the 14th day **(B)** and the 21st day **(C)**.

ERK activation was determined by Western blotting. Compared with the sham group, there were no changes on the expressions of ERK following SCI Figure 6A. However, the OEC administration resulted in a significant increase on the level of p-ERK in the spinal cord both on the 14th day and the 21st day (*P < 0.05) (Figure 6B-6C).

## Discussion

The results show that the OECs from olfactory bulb may induce hyperalgesia when they were transplanted into the hemisected spinal cord in rats. And this phenomenon seemed to be associated with the activation of ERK and the increase of BDNF level, indicated by RT-PCR and Western Blot.

Currently, cell transplantation is one novel therapeutic strategy for the treatment of SCI. As one type of glial cells ensheathed in the olfactory axons, OECs are featured by the function of promoting the axonal regeneration. Several studies emphasized the optimal role of OECs in the treatment of SCI, which may induce function repair for improving locomotor level [8,10-13] and promoting axonal regeneration [14,15], in the paraplegic [10], transected [13,14], contused [8] SCI models and multiple lumbar rhizotomy [16]. Moreover, the introduction of OECs can remyelinate the demyelinated axons in the lesions of the animal spinal cord [17-19]. Unfortunately, however, there is no evidence to reveal how this transplantation affects the sensory function. Therefore, we aim to test whether the treatment of OEC transplantation in rat hSCI model may affect the regulation of sensory function as the motor function.

The innocuous mechanical and thermal stimuli test showed that the OECs treatment group had lower PWL than the untreated group. This result suggests that the OEC transplantation into spinal cord injured rats may induce obvious hyperalgesic responses. For this reason, the analgesic approach should be developed when we consider the OEC transplantation therapy.

To investigate the underlying mechanism of the allodynia following OEC implantation, we determined the expressions of BDNF and ERK. Consequently, BDNF expression in injured spinal cord was greatly increased in OEC-transplanted rats on 21st day and BDNF was located in neurons of the superficial layer in the dorsal horn. It is known that the productions of growth factors (NGF, BDNF, and GDNF) and the expressions of corresponding receptors may contribute to axonal regeneration following OEC transplantation [5]. BDNF as one type of the neurotrophin family of growth factors can not only support the survival of the existing neurons and promote the growth and differentiation of new neurons and synapses [20,21], but also trigger the pain in sciatic nerve separation model [22] and ligation model [23]. In our experiments, immunofluorescent staining was performed to observe the expression of BDNF at the injured segment, and the red fluorescence which symbolized “BDNF positive” was found in the superficial layer of dorsal horn. Simultaneously, the upregulation of BDNF expression was also confirmed. These results indicated that OEC transplantation could increase BDNF expression in sensory neurons of the dorsal horn, which may contribute to the observed phenomena like hyperalgesia.

More importantly, we also found the p-ERK (phosphorylation of extracellular signal-regulated kinase) level was increased in the injured spinal cord after OEC transplantation. The potential correlation of the activation of ERK following spinal neuron injury with the pain hypersensitivity and central sensitization was well documented [24,25]. Within a minute after a noxious stimulus, many p- ERK-positive neurons were observed, predominantly in laminas I and II of the ipsilateral dorsal horn [26], and the phosphorylation of ERK occurring in the nociceptive neurons may contribute to persistent inflammatory and neuropathic pains [27]. In present study, we observed the activation of ERK following OEC transplantation, which may be related to hyperalgesia. In this observation, several events including behavior and molecule have occurred at different time points. Reportedly, the ERK phosphorylation may be a reason for the increase of BDNF [28]. Therefore, the increase of ERK phosphorylation on the 14th day may induce the increase of BDNF level on the 21st day. As the behavioral changes were generally resulting from the cellular and molecular changes, hyperalgesia was found after 1 month. Together, the phosphorylation of ERK results in the upregulation of BDNF, and eventually gives rise to the behavioral hyperalgesia.

We also found that BDNF was expressed both in OECs and in host neurons. As the neurons with BDNF staining are expressing TrkB simultaneously, probably the BDNF released from OECs could be enrolled into neurons by TrkB receptor. These could underline some linkage by BDNF for the consequent hyperalgesia between transplanted OECs and host neurons.

In summary, these findings may provide novel insight into the hyperalgesia responses following OEC transplantation. In using OEC transplantation for the treatment of SCI, it is useful to consider the inhibition of ERK phosphorylation and BDNF blockage to relieve the nociceptive behaviors.

## Conclusion

The transplantation of OECs which can promote axonal regeneration and functional recovery in previous studies may induce noticeable pain hypersensitivity following hSCI. This could be associated with the overexpression of BDNF in the injured spinal cord and the phosphorylation of ERK. The current findings may indicate some therapeutic strategies involving the inhibition of ERK activation or BDNF blockage to treat SCI more correctly and efficaciously in future clinical practice.

## Methods

### Animal and grouping

Adult Sprague–Dawley rats weighing 180–220 g, obtained from the Animal Center of the Sichuan University, were used in this experiment. The animals were divided into three groups randomly (Table 1): Group A rats were underwent hemisected spinal cord injury at the eighth thoracic (T10) cord level, Group B rats were received OECs transplantation after injury, Group C rats were as the sham group (rats were underwent neither SCI nor transplant injections).

**Table 1:** Numbers of animal in each group

**Group**	**PCR**					**Western blotting**		**Behavior and IHC**
	**1st day**	**3rd day**	**14th day**	**21st day**	**28th day**	**14th day**	**21st day**	**28th day**
SCI	5	5	5	5	5	5	5	5
OEC	5	5	5	5	5	5	5	5
Sham	5	5	5	5	5	5	5	5

### Ethics statement

The animal study proposal was legally approved by the Animal Care & Welfare Committee of West China Hospital, Sichuan University with the approval number: 201. All the experiment conforms to the Guide for the Care and Use of Laboratory Animals published by the US National Institutes of Health.

### Isolation, cell preparation and identification of OECs in vitro

The GFP (green fluorescent protein) transgenic mice [29] were sterilized by incubation in the 75% alcohol for 1–2 min, and were sacrificed by decapitation subsequently. Then the head was moved into the culture medium (DMEM with Ham’s F12 - 1:1, Gibco). By using the microscope, the olfactory bulb was dissected and then transported into the cold 10% FBS medium. After washing two times by DMED medium, the tissue block was centrifuged to collect sedimentation. After removing off the supernatant, new DMEM medium was added. By using micropipette, the tissue block was dissected into cell suspension. Then the cell pellets of OECs were placed on a poly-L lysine-coated culture flask at a concentration of 1 × 10^6^/ml. The culture was maintained at 37°C with 5% carbon dioxide in an atmosphere of 95% humidity. After first 12-h incubation, the culture media were transferred into another flask and this process was repeated again for removing the fibroblasts. The culture media containing purified OECs were plated into flask for 7 days, and then were passaged to amplify the numbers of cells till the 3rd passage. To identify the character of cultured OECs, the low-affinity neurotrophin receptor p75NGFR (1:400, Santa Cruz Biotechnology) was used. Briefly, OECs were fixed with 4% paraformaldehyde in 0.1 M phosphate-buffered saline (PBS) for 30 min. For immunohistochemistry, the cells were washed three times in 0.01 M PBS and then were incubated in 3% hydrogen peroxide at room temperature for 20 min in order to block the action of various endogenous peroxidase. After 30 min immersion in PBS (containing 0.3% Triton X-100 and 5% normal goat serum) at 37°C, they were processed for an incubation with anti-rabbit p75 (1:400, Santa Cruz Biotechnology) at 4°C overnight. After washing three times, goat-anti-rabbit IgG was used for incubation for 2 h at 37°C. After washing, they were reacted with DAB in hydrogen peroxide. Following staining for 5 min, the cells were routinely dehydrated. Then the digital images of the cells were photographed and captured with the LEICA automatic photo micrographic system. To determine the purity of OECs, the percentage of P75 positive cells was counted.

### Immunofluorescent staining in vitro

For immunofluorescent staining, the OECs were washed in PBS for 10 min, blocked in 5% normal goat serum (NGS, Sigma), and 0.3% Triton X-100 in PBS for 1 hour at room temperature. Afterward, they were incubated with rabbit anti-p75 (anti-rabbit, 1:400, Santa Cruz Biotechnology) in OECs for 2 h at 37°C and then were incubated overnight at 4°C. After washing three times and the incubation with fluorescent secondary antibody IgG (Goat anti-rabbit CY3: red) for 2 h at 37°C, they were observed under fluorescent microscope. The control cells were incubated in PBS to replace the primary antibody.

### Hemisected spinal cord injury model and administration of ciclosporin A

After anesthetization by intraperitoneal injection of 3.6% chloral hydrate, rats were placed prone on an operating table covered by a warming blanket to maintain the body temperature at 37.0 ± 0.50°C, and then were underwent the laminectomy at the T10 level. The spinal cord was hemisected with microscissors under an operating microscope. The microscissors were inserted into the spinal cord with the tip touching the midline until the left side of the cord was completely divided. To inhibit the immunological rejection, the immunosuppressive agent, known as ciclosporin A (5 mg/ml), was administered intraperitoneally 2 days before cell transplantation. The administration continued (once a day) until the animals were sacrificed.

### Cell transplantation

After cord hemisection, rats were randomly received OECs treatment (n = 5), or normal saline administration (SCI Group, n = 5). And other five rats were underwent neither SCI nor transplant injections (Sham Group, n = 5). Using microscopic visualization, 5 μl OECs at a concentration of 3 × 10^6^/ml were injected into the spinal cord around the injury site. The sham group rats were underwent laminectomies only without SCI or transplant injections. After injection, the superficial back muscles and the skin were sutured along the midline. All the operated rats were allowed to recover spontaneously, besides the administration of penicillin for 3 days.

### Behavior test: the nociceptive sensory assessment

One month after cells injection, sensation of each group on the mechanical and thermal stimulation was assessed.

Hind paw sensitivity to innocuous mechanical stimulation was evaluated by ANALGESY METER (UGO BASILE, Italy, 37215). According to the previous description of Randall and Selitto [30], the nociceptive thresholds (also called paw withdrawal threshold, PWT), expressed in grams, were measured by applying an increasing pressure to the hind paw until withdrawal. The cut-off point was set to 500 g for preventing the damage.

Thermal hyperalgesia was measured by the Plantar Analgesia Meter (UGO BASILE, Italy, 7370) according to the method described by Hargreaves et al. [31]. Briefly, the rats were placed in transparent plastic enclosures on a glass plate, then acclimatized to test environment for 1 hour. A fixed intensity (intensity 80) and movable radiant heat source was placed underneath the plate and focused onto the plantar surface of each hind paw. The nociceptive endpoint was obvious withdrawal of the hind paws and the paw withdrawal latency (PWL) was recorded. An automatic 20 s cutoff was used to prevent tissue damage.

### Sample harvested and observation of OECs in vivo

After measurement of sensory function, all rats for immunohistochemistry were sacrificed and the spinal cords were harvested. Spinal cords were sectioned by using microtome (Leica, Germany) into 20 μm thickness sections. To detect the survival of OECs in host spinal cord, the OECs derived from GFP transgenic mice around the lesion site in the spinal cord was observed by immunofluorescent microscope. The samples for RT-PCR were harvested from different time point on the 1st, 3rd, 14th, 21st, 28th day after the operation or OECs administration; while the spinal cords for Western Blot were collected from the time point on the 14th day and 21st day.

### Immunofluorescent observation and double labeling in vivo

To detect the survival of OECs in the spinal cord, the spinal cords were sectioned (20 μm), and then observed under fluorescent microscope. To detect whether or not transplanted OECs could express BDNF, and the host neurons in the spinal could express BDNF and its functional receptor TrkB, we performed immunofluorescent staining. Briefly, sections were blocked with 3% goat serum in TBS, then incubated overnight with anti-TrkB (mouse, 1:400, MILLIPORE), anti-NeuN (mouse, 1:500, MILLPORE), or anti-BDNF (rabbit, Chemicon, 1:500) at 4°C. After washing three times, secondary antibodies conjugated to the fluorescent markers (anti-rabbit Cy-3, red light, 1:100; anti-mouse 488: green) were used for 24 h incubation at 4°C. Lastly, the sections were observed under fluorescent microscope after washing three times. The control sections were incubated lonely in PBS, and there is no positive staining.

### RT-PCR

The tissues from the injury segments of OECS group, operated group, and sham group were collected and homogenized, respectively (the 1st, 3rd, 14th, 21st, 28th day). The supernatant was obtained from the spinal cord of each group, followed by a centrifugation (12,000/g). By using Nanodrop spectrophotometer (ND-1000), the concentrations of RNA samples were measured. The equal amount of RNA (4lg) was used for each experiment. Using the β-actin as the internal control, the RT-PCR technique was performed to determine the level of BDNF mRNA. Gene primers were synthesized by TaKaRa Company (Japan). The primer sequences for the upstream and downstream of BDNF are respectively: 5′ TCCCTGGCTGACACTTTT 3′, 5′ ATTGGGTAGTTCGGCATT 3′ (the bp is 466 bp), while for β-actin is : 5′GTAAAGACCTCTATGCCAACA 3′, 5′ GGACTCATCGTACTCCTGCT 3′(the bp is 227 bp). By using Revert AidTM First Strand cDNA Synthesis Kit (Fermentas, USA), the first strand cDNA synthesis was prepared from 4 μg of total RNA in order to amplify RNA. Then the PCR was performed with the PCR MasterMix Kit (Fermentas, USA). The whole process for 30 cycles included denaturation for 1 min at 94°C, annealing for 1 min, and extension for 1 min at 72°C. Subsequently the products of PCR were electrophoresed in 1% agarose gel, stained with ethidium bromide, and then visualized by ultraviolet gel imager (BioRad, USA). The optical density (OD) of each product band which consists of objective gene and β-actin was obtained, and then the OD ratio between objective genes and β-actin were calculated to quantify the relative OD values.

### Western blot analysis

The tissues from the injury segments in OECs group, operated group and sham one on the 14th day, and the 21st day were harvested respectively. After homogenized with ice-cold PBS and lysed for 5 min in lysis buffer (Pierce), the tissues were centrifuged (12,000/g), the supernatant was collected. The protein concentrations of the supernatants were tested by applying the micro bicinchoninic acid (BCA) method. Equal amounts of protein (20 μg) were denatured by boiling 5 min in Laemmeli buffer, separated by 12% SDS-polyacrylamide, and transferred onto nitrocellulose membrane. And the membranes were blocked with low-fat milk in Tris-buffered saline and incubated with primary antibodies against rabbit p-ERK (1:500, cell signal) or ERK (1:1000, cell signal). After incubating with HRP-conjugated secondary antibodies (goat anti-rabbit IgG, 1:5000), the labeling was detected using chemiluminescence reagents. To determine the level of ERK and p-ERK, β-actin was used as internal control. All pictures were collected and the bands were measured by expressing as optical densities. Then the target band and β-actin ratio was counted and used for the statistic analysis.

### Statistical analysis

Quantitative variations were evaluated by mean ± SD. Data were analyzed using one-way ANOVA by SPSS software package after a suitable correction. The statistical significance was defined as P < 0.05.

## Competing interests

The authors declare that they have no competing interests.

## Authors’ contributions

BCL participated in the design of the study, performed hemisected spinal cord injury model, performed the behavior test, performed the statistical analysis, and drafted the manuscript. ZZ performed the isolation, cell preparation, and identification of cells, performed immunofluorescent staining, and drafted the manuscript. LYL performed hemisected spinal cord injury model, performed cell transplantation and performed sample harvest. TYW performed RT-PCR, and performed Western Blot analysis. JL provided useful discussion, and participated in the design and coordination of the study. LHY provided useful discussion, and conceived of the behavior test. DQL provided useful discussion and participated in the study. WSZ conceived of the study, participated in the design and coordination of the study and drafted the manuscript. THW conceived of the study, participated in the design and coordination of the study, performed statistical analysis and drafted the manuscript. All authors read and approved the final manuscript.
